# 

**Published:** 1880-06

**Authors:** 


### THE MILLERS.

An international convention of millers is being held in this city and will continue about a month.

The subjects that will come before this body are of vital importance to all people, viz., how shall our bread making materials be best prepared for use? To what processes shall the grain be subjected to yield the largest amonnt of nourishing, health-giving bread?

These are two important points; the latter more so than the former. The consideration of the kinds, qualities and conditions of bread-making grains will be in order. The methods of converting the grain into flour or meal will afford important matter for discussion and investigation. In this country, up to this time, there has been but little, if any, associated effort in behalf of this great and universal interest.

The testing and comparing methods, appliances and machinery results in improvement, and tends to perfection.

The common methods of converting the grain into flour has often been criticised by physicians and dentists. The points discussed, by the latter more particularly, is the almost entire removal of the bone-making material of the grain in the process of grinding, and so reducing the nutrient quality of the bread, so far at least as the bones and teeth are concerned. That these in many instances suffer greatly from a want of proper supply of nutriment material, especially during the period of growth and development, there is no doubt.

In the preparation of fine flour the earthy material, which is almost exclusively in the covering of the grain, is separeted and removed from the inner part, instead of being ground and commingled with it. The retention of the outer part of the grain, in part at least, of the flour, would add considerably to its nutrient quality.

The bread of the world is one of its great interests, one in which every human being of the civilized world is interested; one that touches our physical comfort and well-being as definitely and sharply as any other. All should embrace the opportunity of visiting this great display. Its equal, in this direction, has never been seen in the world before, and may not again for a long time, and none who can make the opportunity available should miss it.

### CONNECTICUT VALLEY DENTAL SOCIETY.

The semi-annual meeting of the Connecticut Valley Dental Society for 1880, willl be held at the Sea-View House, Savin Rock, West Haven, Connecticut, on Thursday and Friday, June 17th and 18th.

The Executive Committee in their selection of this beautiful place on the Sound, feel that it is much more central and eligible than some that were suggested to them. It is certainly a spot where both pleasure and profit may be obtained at this time by every dentist who can attend. It is distant from New Haven but three and one-half miles; accessible by horse cars and boat. The hotel accommodations are ample and excellent, and the rates will be reduced to dentists and manufacturers to $2 per day. The horse cars leave the railroad depot at New Haven every fifteen minutes for Savin Rock, passing the entrance to the Sea View House.

Let every dentist, whether in membership or not, be present. All manufacturers and dealers are also invited to be present.

ESSAYISTS.

George W. Lovejoy, M. D., Montreal, P. Q.; "Dental Status of the Dominion of Canada." Robert A. Fones, D. D. S., Bridgeport, Conn., "Dental Hygiene." Newton Morgan, D. D. S., Springfield, Mass., "The use of Confectionery and its Influence upon the Teeth." It is expected that interesting clinics will be given at intervals between sessions. Subjects for discussion: "Incidents of Office Practice." "Dental Legislation." Those having volunteer essays will please to notify the chairman of the executive committee on or before the opening of the meeting.

A. M. Ross, Secretary.

Chicopee, Mass., May 10th, 1880.

### MEETING.

The tenth annual session of the New Jersey State Dental Society will be held at Long Branch, July 20th, 21st and 22nd. The Board of Examiners will meet on the 19th inst. for the examination of candidates for license to practice. The profession generally are invited to be present, Long Branch being easy of access, with unsurpassed hotel accommodations. Every attention will be given inventors and dealers in dental appliances for the proper exhibition of their goods. Those so desiring will communicate with Dr. J. C. Clarke, chairman executive committee, Jersey City, N. J.

Chas. A. Meeker, Secretary.

The following persons have been appointed delegates to represent the Mississippi Valley Dental Association in the American Dental Association at Boston, the first Tuesday of August: S. J. Way, J. W. Jay, Otto Arnold, E. G. Betty, H. L. Moore, J. E. Cravens, J. F. Dennis, J. G. Cameron, T. S. Hacker, C. J. Keeley, T. S. Brown, Wm. Taft, W. Storer How, D. C. Whaley, C. R. Taft, A. Berry, F. W. Sage, J. S. M. Campbell, A. G. Rose, A. Taft.

J. Taft,<br>

N. S. Hoff, } *Committee.*

### CALIFORNIA STATE DENTAL ASSOCIATION.

The eleventh annual session will be held in San Francisco, commencing Tuesday, June 8th, 1880, at 10 o'clock a. m., and continue four days.

SUBJECTS.

Treating and filling pulpless teeth, A. Warner, Jr. The relation between dental surgery and medicine, W. A. Knowles. The management of deciduous teeth, S. E. Knowles. Dental etiology, W. J. Younger. Treatment of the mouth preparatory to filling, T. S. Whipple. Dental hygiene, H. C. Davis. Alimentation of tooth tissues, W. H. Robinson. The new departure, W. F. Griswold.

All papers, of whatever character, that are to become the property of the Association should be so carefully prepared *before* their presentation, that they may pass into the hands of the Publication Committee and printer without change.

That all papers may be uniform, use legal cap and write on one side only. By so doing you will save much valuable time, annoyance and labor to the Publication Committee and Secretary.

### AMERICAN DENTAL ASSOCIATION.

The American Dental Association will hold its twentieth annual session, August 3d, 4th, 5th and 6th, 1880, at the hall of the Massachusetts Institute of Technology, Boston, Mass. Hotel accommodations at the "*Brunswick*," Boylston, corner Clarendon streets, opposite the Institute. Rates, $3 per day, reduced from $4.50. This hotel is new and first-class in every appointment. The hotel and hall are in near proximity to the business part of the city, yet free from the noise and turmoil necessarily incident to the business streets. The committee of arrangements feel sure they will be able to provide opportunities to make the stay of the members pleasant and the meetings of the association profitable.

Thos. Fillebrown, Chairman 1st Div. Ex. Com.

### THE DENTAL REGISTER,

A MONTHLY MAGAZINE DEVOTED TO THE INTERESTS OF

**THE DENTAL PROFESSION.**

*TERMS REDUCED TO $2.50 PER YEAR. SINGLE COPIES 25C.*

EDITED BY PROF. J. TAFT, D.D.S.

AND

*Published by SPENCER, CROCKER & CO., Ohio Dental Depot.*

The volume commences in January and closes in December.

The REGISTER being issued monthly is always fresh, therefore Indispensable to the practitioner who desires to keep posted up with the new inventions and improvements which are daily developed. Neither expense nor effort will be spared to give value and variety to its contents. Articles will be illustrated with well executed engravings whenever they will add to the interest or assist to explanation.

Its extensive circulation and frequency of issue make it one of the BEST DENTAL ADVERTISING MEDIUMS in the United States.

All subscriptions must begin with January or July.

Local or special notices, five lines or less, will be inserted for$3 each insertion; over five lines, 50 cts. per line.

All advertising bills payable in advance, or at furthest, after the issue of the first number containing the advertisement. All transient advertisements to be paid for in advance.

☞CONTRIBUTIONS SOLICITED.

Exclusively Editorial Communications should be addressed to Editor.

The latest number of the REGISTER may be ordered with or without other goods at any time, and all letters should be addressed to

*SPENCER, CROCKER & CO., Ohio Dental Depot, Cincinnati, O.*

